# Diffraction-limited ultrabroadband terahertz spectroscopy

**DOI:** 10.1038/srep24811

**Published:** 2016-05-04

**Authors:** M. Baillergeau, K. Maussang, T. Nirrengarten, J. Palomo, L. H. Li, E. H. Linfield, A. G. Davies, S. Dhillon, J. Tignon, J. Mangeney

**Affiliations:** 1 Laboratoire Pierre Aigrain, Ecole Normale Supérieure, CNRS (UMR 8551), Université P. et M. Curie, Université D. Diderot, 75231 Paris Cedex 05, France; 2 School of Electronic and Electrical Engineering, University of Leeds, Woodhouse Lane, Leeds LS29JT, UK

## Abstract

Diffraction is the ultimate limit at which details of objects can be resolved in conventional optical spectroscopy and imaging systems. In the THz spectral range, spectroscopy systems increasingly rely on ultra-broadband radiation (extending over more 5 octaves) making a great challenge to reach resolution limited by diffraction. Here, we propose an original easy-to-implement wavefront manipulation concept to achieve ultrabroadband THz spectroscopy system with diffraction-limited resolution. Applying this concept to a large-area photoconductive emitter, we demonstrate diffraction-limited ultra-broadband spectroscopy system up to 14.5 THz with a dynamic range of 10^3^. The strong focusing of ultrabroadband THz radiation provided by our approach is essential for investigating single micrometer-scale objects such as graphene flakes or living cells, and besides for achieving intense ultra-broadband THz electric fields.

Terahertz spectroscopy in the time-domain is widely used for fundamental investigations of condensed and biological matter as well as for a broad range of applications. Owing to the increased availability of ultra-short pulse lasers (<20 fs), THz time-domain spectroscopy (TDS) systems extending up to few tens of THz are progressively being developed providing full complex THz and mid-infrared electric field measurements as well as new insights on dynamics occurring faster than a single oscillation cycle of light^1^. Ultra-broadband THz pulses are mostly generated by photoionization of gas plasmas^2^, optical rectification in nonlinear crystals^3^ or ultrafast photoconduction in antennas^4^. A great challenge of ultrabroadband THz spectroscopy systems is to reach the ultimate diffraction resolution limit over the multi-octave spanning frequency. Indeed, THz generation in gas plasmas shows a conical far-field distribution depending on the THz frequency, which prevents diffraction-limited focusing using classical optical arrangements^5^. By exploiting the highly spatially confined THz beam that propagates inside the filament^6^, J. Zhao *et al.* have recently achieved a TDS system with sub-diffraction limited resolution but covering only a narrow THz spectral range. Furthermore the radiation diagram of ultra-broadband THz pulses emitted by optical rectification in nonlinear crystals strongly varies with the THz frequency due to the involved diffraction regimes^7^. To approach the ultimate λ^3^ confinement of THz pulses generated by nonlinear crystals, M. Shalaby *et al.*^8^ and H. Hirori *et al.*^9^ have employed concepts based on pump-pulse divergence control. However, the proposed methods are sophisticated or require iterative optimization procedures and the diffraction-limited focusing of the THz radiation was not demonstrated over a broad spectral range. As for photoconductive antennas (PA) employed as emitter in ultrabroadband THz TDS system^10^,^11^, they deliver THz radiation with a frequency dependent divergence, making focusing of the multi-octave THz pulses to reach diffraction-limited resolution a challenging issue^12^. Beck *et al.*^13^ and A. Leitenstorfer *et al.*^14^ have previously demonstrated the focusing of the THz radiation delivered by PA by tailoring of the exciting laser beam, but their techniques cannot be directly implemented in conventional THz TDS systems.

Here, we present an original concept based on wavefront manipulation to achieve ultra-broadband THz TDS system with diffraction-limited resolution. This easy-to-implement concept relies on spherical wavefront optical excitation of the emitter providing an ultrabroadband THz emission with frequency-independent divergence and wavefront. By applying this concept to a large-area PA emitter, we experimentally demonstrate an ultra-broadband TDS system with diffraction-limited resolution up to 14.5 THz and a dynamic range of 10^3^.

In THz TDS systems, THz emitters such as large-area PAs are commonly excited by femtosecond optical pulses with plane-wavefront. Indeed, either a collimated or low-divergent optical excitation beam is used or the THz emitter is placed close to the beam waist of a divergent optical excitation beam^7^,^15^,^16^. Under plane-wavefront optical excitation, the propagation properties of the THz radiation emitted by large-area PAs are governed by two distinct diffraction regimes according to the spectral range. For λ_THz_ > 2π*w*_*THz*_, where *w*_*THz*_ is the THz beam radius on the surface of the PA, THz radiation diffracts in all directions leading to a strong divergence^17^,^18^, as expected by Bethe’s theory of diffraction by small holes^19^. For λ_THz_ < 2π*w*_*THz*_, the propagation characteristics of the THz radiation follow Rayleigh diffraction. In this regime, the wavefront of the THz radiation at the surface of the antenna is imposed by the plane wavefront of the optical pump beam. Thus, the THz radiation behaves as a paraxial Gaussian beam with a beam waist located at the surface of the emitter and with a radius *w*_*THz*_ that is constant for all frequencies. Owing to the wavelength conversion from the optical to THz spectral range, the emitted THz electric field strongly diverges following the frequency-dependent relation 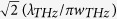. Since the THz electric field emitted by the large-area PA is proportional to the optical pump intensity, the THz waist radius *w*_*THz*_ is expressed as 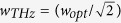, where *w*_*opt*_ is the optical spot radius on the surface of the large-area PA. The calculated propagation properties of THz radiation emitted by a large-area PA under plane-wavefront optical excitation are illustrated Fig. 1a,b at selected frequencies higher than c/(2π*w*_*THz*_). In THz TDS systems, two 90° off-axis parabolic metal mirrors are usually employed for collecting and subsequently focusing the emitted THz radiation, usually located at the focus of the collecting parabolic mirror. At its focal point, for components that verify λ_THz_ < 2π*w*_*THz*_, the focusing mirror produces a virtual image of the illuminated surface of the THz emitter excited by plane-wavefront optical excitation. Thus, the waist radius of the focused THz beam remains constant for all frequencies and extends over *w*_*THz*_, as illustrated Fig. 1c. Consequently, the resulting spatial resolution of the broadband TDS system is poor compared to diffraction-limited TDS systems. However, achieving diffraction-limited focusing of the THz beam is a non-trivial task since it requires THz emission with frequency-independent divergence and wavefront (over more than 5 octaves).

Our original approach to achieve THz emission with frequency-independent divergence and wavefront exploits the control of the THz radiation propagation properties by the spatiotemporal profile of optical excitation pulses. Indeed, THz emission from photoconductive antennas with typical dimensions larger than the characteristic wavelength of the radiation can be decomposed in Fourier space as a superposition of plane waves, which is triggered by the arrival time of the optical pulses on the emitter. Thus, the wavefront and divergence of the emitted THz radiation are controlled by the spatially dependent THz amplitude at the surface of the emitter and by the spectral phase^20^ (see Supplementary Information). The spatially dependent THz amplitude at the surface of uniform emitters that convert optical pulse excitations into THz electric field is determined by the optical pump pulse intensity profile. As for the spectral phase, it is determined by the spatially dependent optical delay in excitation. Our optical excitation scheme consists in illuminating the THz emitter with a strong divergent optical beam and placing it outside of the Rayleigh zone of the optical excitation beam, so that the optical pulses are incident on the emitter with a spherical wavefront. A spherical-wavefront can be achieved just by focusing a collimated optical beam with a lens. However, for ultrashort optical pulses, parabolic mirrors are preferred over lens to avoid any temporal distortion of the optical pulses. As a result, the wavefront of the divergent optical beam in our scheme is close to spherical but with a slight asymmetry owing to the use of an off-axis parabolic mirror. This spherical-wavefront excitation scheme is illustrated in Fig. 2a (left) for a forwards collection of the THz radiation and in Fig. 2a (right) for a backwards collection of the THz radiation. Therefore, the wavefront of the THz beam at the surface of the PA is spherical, imposed by the optical excitation wavefront.

We first numerically investigate the propagation properties of the THz radiation emitted by a large-area PA under this original excitation scheme relying on spherical-wavefront optical pulses. Here, we apply the Huygen’s principle of superposition of spherical wavelets to calculate the propagation along *z* direction of the electric field *E*_*THZ*_, radiated by a square antenna of dimensions L × L = 0.5 × 0.5 mm^2^ and located in the plane (*x*, *y)*. We consider an optical excitation beam with a divergence angle of 0.25 radian, a Gaussian spatial intensity profile and a beam radius at the surface of the PA, *w*_*opt*_ = L/2. The wavefront of the optical excitation beam considered in the simulation is spherical. Figure 2b shows the calculated spatial profiles along x direction of *Re*(*E*_*THz*_) in the far field at 4 THz and 10 THz. One can see that whereas the frequency is increased by a factor ×2.5, the divergences of the emitted THz beams are similar. Moreover, the THz radiation fully conserves the phase front information of the optical pump beam during its propagation. The calculated divergence of |*E*_*THZ*_| respected to the frequency is reported in Fig. 2c. For spectral components lower than 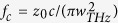, corresponding in our case to *f*_*c*_ = 3 THz (see Supplementary Information), the divergence of |*E*_*THZ*_| decreases as the frequency increases owing to the negligible effect of the curvature of the optical beam and does not follow anymore the divergence of a Gaussian beam of waist radius *w*_*THz*_ (red lines) but becomes frequency-independent. More quantitatively, the divergence of |*E*_*THZ*_| is equal to 0.25 radian, i.e. the divergence of the optical intensity, despite the three orders of magnitude difference in wavelength. We have verified that this equality is preserved for any *w*_*THz*_, *w*_*opt*_ and L until *f*_*THz*_ > *f*_*c*_. Thus, for all frequencies, the emitted THz radiation resembles a paraxial Gaussian beam with a divergence and transverse section of the electric field in the far field equal to the beam divergence and the beam radius of the optical excitation beam. Considering Gaussian beam propagation, the THz radiation behaves as a Gaussian beam with a beam waist virtually located before the position of the large-area PA. At this position, the virtual THz beam radius is expressed as 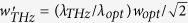 (see Supplementaty Information) and is drastically larger (more than two order of magnitude) than the optical waist radius. In other words, the wavefront of the emitted THz radiation corresponds to the wavefront that would be delivered by a virtual single THz emitter located at the focus of the collecting parabolic mirror. These results demonstrate that our concept based on spherical-wavefront optical excitation provides THz emission with frequency-independent divergence and wavefront until *f*_*THz*_ > *f*_*c*_, which is a promising feature to achieve diffraction-limited focusing of the THz radiation in conventional TDS systems.

We now provide an experimental validation that our concept relying on spherical-wavefront optical excitation of a THz emitter enables diffraction-limited focusing of the THz radiation. For this purpose, we develop a THz TDS system implementing the spherical-wavefront optical excitation scheme (see details in Supplementary Information). Our folded parabolic mirror configuration for the optical focusing parabolic mirror and the THz collecting parabolic mirror ensures the correction of the asymmetric wavefront induced by the use off-axis parabolic mirrors. Consequently, the THz radiation is collimated when incident on the THz focusing mirror. We process a large-area interdigitated PA device^18^ of 0.5 × 0.5 mm^2^ dimensions made from low-temperature grown GaAs material, which should provide strong THz emission at high frequencies compared to semi-insulating GaAs active layer^21^. The TDS setup uses a 15 fs, 80 MHz repetition rate pulse train centered at a wavelength of 800 nm delivered by a Ti:sapphire oscillator and a pulse compressor. The large-area PA is illuminated by optical pulses of energy 4 nJ, focused by a parabolic mirror with a numerical aperture of 0.25 radian (f-number of 2). The THz radiation emitted by the large-area PA is collected backward to avoid absorption and dispersion of the THz pulses in the GaAs wafer. We examine the focusing of the THz radiation emitted by the large-area interdigitated PA, after being reflected by two off-axis parabolic mirrors above 3 THz (i.e. for *f*_*THz*_ > *f*_*c*_). To characterize the THz spot at focal position of the focusing mirror, we employ the knife-edge technique that consists in measuring the partially cut propagated field induced by a razor blade^22^. A conventional electro-optic detection scheme is used for probing the THz field in a 20 μm-thick ZnTe crystal after being propagated by two other off-axis parabolic mirrors. The optical beam size on the electro-optic crystal is 90 μm. In first approximation, we assume that the focused THz beam is symmetric in the transverse plane. The Fourier transform of the experimentally acquired time-domain waveforms were computed to extract the spectral amplitude |*E*(*ω*)| at each frequency for each position of the knife-edge. Figure 3a shows the spectral amplitudes for four representative frequencies. One can see that the slope of the knife-edge profiles increases as the THz frequency increases. By performing a spatial derivative of the measured knife-edge profiles for each frequency, we evaluate the spatial distribution of |*E*(*ω*)| along the horizontal transverse direction, we extract the waist radius *w*_*THz*_ of |*E*(*ω*)| and the full-width at half maximum (FWHM) of the intensity of the THz radiation, *I*(*ω*) at the focus of the focusing mirror (see Fig. 3b, black squares). The waist radius, defined by the distance from the beam axis where |*E*(*ω*)| drops to 1/e of its maximum value, significantly decreases as the THz frequency increases and is reduced down to 60.8 μm at 14.5 THz. Note that smaller waist radius could be achieved with lower f-number focusing parabolic mirror. The theoretical FWHM of a Gaussian beam profile at the diffraction limit is plotted (given by 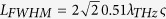 with *ς* the f-number of the focusing parabolic mirror) in Fig. 3b (red curve) and we observe an excellent agreement for components up to 8 THz. From 8 THz to 13 THz, a very slight deviation is observed, which is possibly from the additional THz signal delivered by the GaAs LO phonon emission at 8.7 THz. The experimental data tends to the diffraction limit above 13 THz. These results clearly demonstrate the wavelength-square confinement of the THz energy under spherical-wavefront optical excitation scheme over an ultrabroad spectral range up to 14.5 THz. We then compare theses results to the spatial distribution of |*E*(*ω*)| under plane-wavefront optical excitation scheme. For these measurements, the experimental conditions are similar except that the incident optical pump beam is almost collimated when exciting the large area PA. In these conditions, the signal-to-noise ratio of the THz signal is reduced at high frequencies compared to the spherical-wavefront optical excitation condition, limiting the achievable upper frequency to 9.5 THz. The waist radius *w*_*THz*_ and the FWHM of *I*(*ω*) at the focus of the focusing mirror extracted under this plane-wavefront excitation scheme are constant for all components (see Fig. 3b, blue squares). We can deduce that the focused THz spot area at 14.5 THz is 8.5 times smaller under spherical optical excitation than under plane-wavefront optical excitation scheme (dashed line). Our model reproduces all the experimental features: the knife-edge profiles and the spectral dependence of the THz electric field waist radius under both optical excitation schemes.

The tight focusing of THz energy provided by the spherical-wavefront optical excitation scheme enhances the sensitivity of the electro-optic sampling detection. Indeed, the time-dependent electro-optic signal is expressed as a Fourier integral that involves the product of the autocorrelation of the optical probe field and the THz electric field in the electro-optic crystal^23^. Thus, since the strong confinement of the THz radiation onto the electro-optic crystal enhances the THz electric field amplitude, the electro-optic signal is increased. Figure 4a shows the temporal waveform of THz radiation emitted. The temporal waveform shows a bipolar shape followed by oscillations. The initial negative transient and the subsequent positive transient have FWHMs of 100 fs and 273 fs, respectively. The amplitude spectra under spherical-(blue line) and plane-(red line) wavefront optical excitation, obtained by Fast-Fourier transform are shown in Fig. 4b. Under spherical-wavefront optical excitation, the amplitude spectrum is maximum at 1.5 THz, extends up to 22 THz and the dynamic range is 10^3^, (i.e. 60 dB in power). The bandwidth and the dynamic range are 1.4 and 10 times higher respectively than in the previous report based on large area interdigitated PA based on SI GaAs active layer at similar pulse energy of 4 nJ^15^. This result highlights the emission of additional high-frequency components provided by the LTG GaAs material and the stronger signal achieved by the spherical-wavefront optical excitation scheme. The comparison between the spectra obtained under these two optical excitation schemes clearly highlights the enhancement of the TDS system dynamic range provided by our approach (insert Fig. 4b). Indeed, above 3 THz, the spectral amplitude is significantly increased under spherical-wavefront optical excitation scheme and higher is the frequency, higher is the spectral amplitude enhancement.

We now investigate experimentally the detected spectral amplitude as a function of the optical probe size over the full spectral range. Since the THz radiation is focused onto the electro-optic crystal down to the diffraction limit, a spectral dependence of the THz detected signal is expected as a function of the optical probe size in contrast with standard THz TDS system. We measure the temporal waveforms detected with different optical spot radius ranging from 410 μm to 90 μm and report in Fig. 4c the spectral amplitude measured with 280 μm, 175 μm and 90 μm optical probe radius normalized by the spectral amplitude measured with 410 μm optical spot radius above 4 THz. As the optical probe radius is reduced, the dynamic range is increased. Reducing the optical probe radius from 410 μm to 90 μm enhances the signal to more than a factor 6 at 14 THz. We will further reduce the optical probe size by using lower f-number parabolic mirror for focusing the optical probe beam.

Our approach can be applied to various uniform and large THz emitters that convert optical pulse excitations into THz electric field. For instance, in TDS systems that use a centimeter-size PA excited by a regenerative amplifier laser as a emitter, this spherical-wavefront excitation scheme remains easily implementable since illuminating the whole area of the PA only requires a modification of the PA position along the optical excitation beam path. A diffraction-limited THz focus cannot be achieved over the whole spectral range using the usual low-divergent optical excitation scheme with a single parabolic mirror to focus the THz radiation, since the low divergence of the emitted THz radiation is frequency-dependant. In addition, in TDS systems that use nonlinear crystals as emitters (such as ZnTe or GaP), our concept remains applicable since the THz waves are radiated by a dipole radiation process in the nonlinear crystal with an amplitude proportional to the intensity of the laser pulse^24^.

## Conclusion

In conclusion, we experimentally and theoretically demonstrate diffraction-limited ultra-broadband TDS from up to 14.5 THz, relying on spherical-wavefront optical excitation of the THz emitter. This extremely easy-to-implement concept applied to a LTG GaAs large-area interdigitated PA emitter provides an ultrabroadband THz TDS system with up to 22 THz bandwidth associated with a dynamic range of 10^3^. Our work represents a strong support for the development of advanced ultra-broadband THz TDS systems that is currently growing owing to the proliferation of 15-fs pulse lasers. Furthermore, this work opens up new avenues for THz investigations of single micrometer-scale objects in a wide range of science areas, such as graphene flakes or living cells. This work also has the potential to greatly impact nonlinear THz applications as it can provide intense ultra-broadband THz electric field at the diffraction limit.

## Additional Information

**How to cite this article**: Baillergeau, M. *et al.* Diffraction-limited ultrabroadband terahertz spectroscopy. *Sci. Rep.* **6**, 24811; doi: 10.1038/srep24811 (2016).

## Figures and Tables

**Figure 1:**
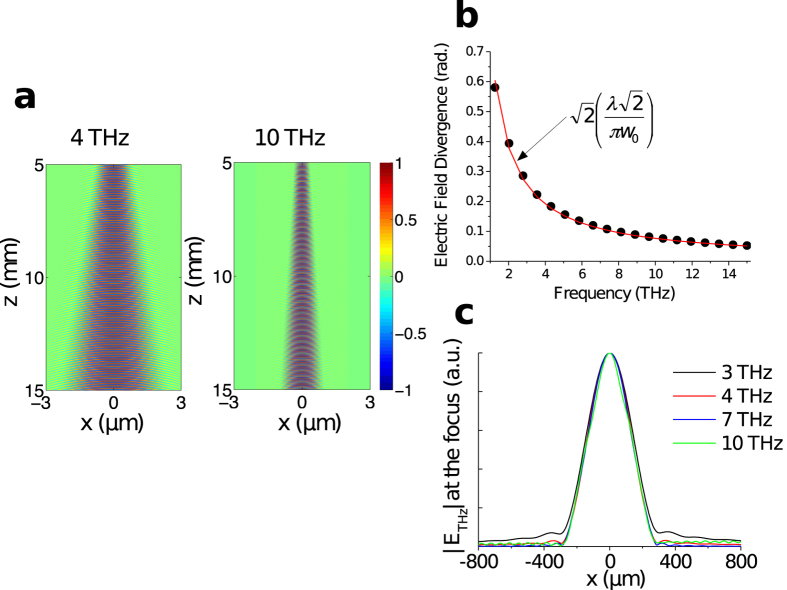
THz radiation properties under plane wave-front optical excitation of the emitter at selected frequencies higher than c/(2π*w*_*THz*_). (**a**) The normalized calculated spatial profiles of *Re*(*E*_*THz*_) along x direction in the far field (from 5 mm to 15 mm away from a PA) at 4 THz and 10 THz. (**b**) The calculated divergence angle of |*E*_*THz*_| emitted by a PA with dimensions 0.5 × 0.5 mm^2^ as a function of frequency (black circles). The red line is divergence angle of |*E*_*THz*_| considering a Gaussian beam with waist radius *W*_*THz*_. (**c**) The normalized distribution of the |*E*_*THz*_| at the focus of the focusing parabolic mirror for different frequencies for TDS system with magnification of 1.

**Figure 2:**
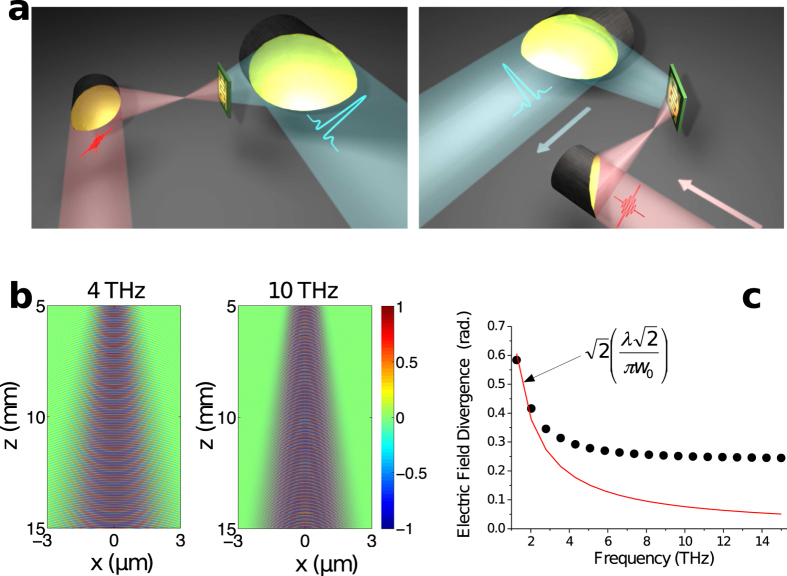
THz radiation properties under spherical wave-front optical excitation of the emitter. (**a**) Schematic of the concept of a THz emitter excited by a spherical wavefront optical pulse in a reflected (left) and transmitted (right) configuration: the emitter (here a large-area PA) is both illuminated by a divergent optical pump beam, the divergence angle of the optical pump intensity is chosen to match the numerical aperture of the collecting parabolic mirror. The emitter is positioned after the focus of the optical excitation beam, at a distance such that the whole surface of the emitter is illuminated without light loss. For a square antenna with a side length L, the optimum position is at a distance z_0_ (see Supplementary information) just after the focus of the optical excitation beam. (**b**) The normalized calculated spatial profiles of *Re*(*E*_*THz*_) along x direction in the far field (from 5 mm to 15 mm away from a PA) at 4 THz and 10 THz. (**c**) The calculated divergence angle of |*E*_*THz*_| as a function of frequency for a PA of size L × L = 0.5 × 0.5 mm^2^. The red line is the divergence angle of |*E*_*THz*_| considering a Gaussian beam with waist radius *w*_*THz*_.

**Figure 3:**
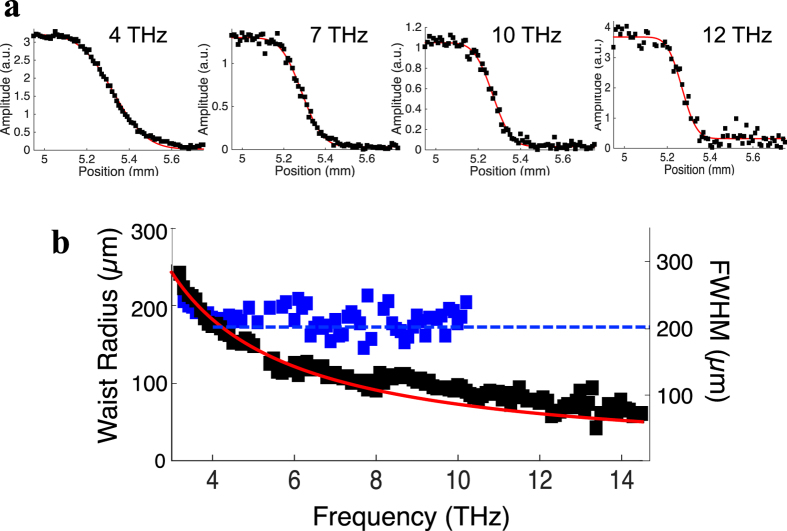
Experimental characterization of the THz radiation at the focus of the focusing mirror. (**a**) Spectral amplitudes of the THz radiation against the transverse position of the knife-edge at the focus normalized to their maxima for different frequency (4 THz, 7 THz, 10 THz and 12 THz). The black squares are the experimental data and the red lines represent the fitting of data by the error function. (**b**) Spectrally resolved waist radius *w*_*THz*_ of |E(ω)| and the full-width at half maximum of I(ω) at the focus of the focusing mirror estimated from the knife-edge measurements under spherical wavefront optical excitation (black squares) and under plane wavefront optical excitation (blue squares). The dashed blue line represents the predicted waist radius under plane wavefront optical excitation. The red curve indicates the FWHM of a Gaussian beam profile at the diffraction limit for a focusing parabolic mirror with a f-number of 2, corresponding to the f-number value of the focusing parabolic mirror used in our experiment.

**Figure 4:**
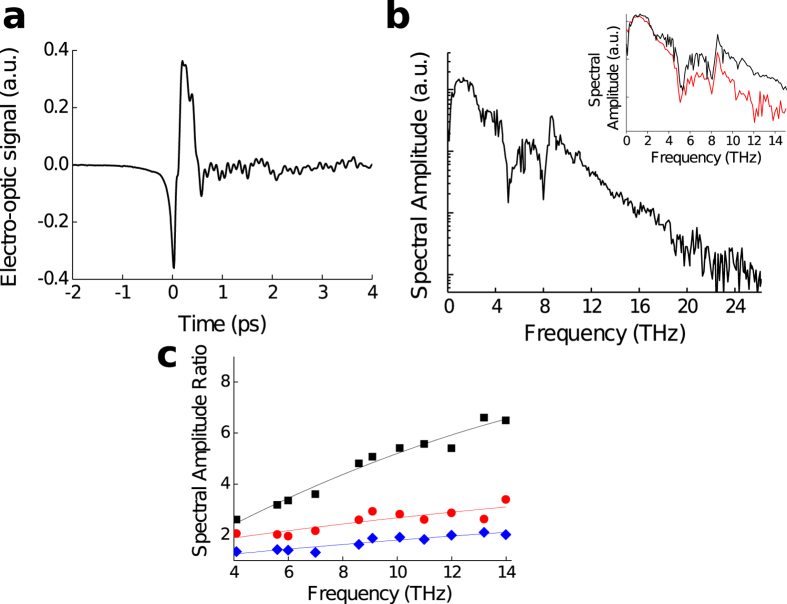
Performances of our TDS system exploiting the concept of spherical-wavefront optical excitation. (**a**) Temporal waveform emitted by the LTG GaAs interdigitated PA under spherical wavefront optical excitation at 4 nJ, and the corresponding amplitude spectra (**b**). The spectrum shows distinct features corresponding to TO-phonon absorptions of ZnTe at 5.1 THz and GaAs at 8.0 THz and also the phonon emission peak at 8.7 THz corresponding to the LO phonon oscillations in GaAs. Insert : Amplitude spectra measured under spherical-(blue line) and plane-(red line) wavefront optical excitation (**c**) Spectral amplitude measured with 280 μm, (blue diamonds), 175 μm (red circles) and 90 μm (black squares) optical probe radius normalized by the spectral amplitude measured with a 410 μm optical spot radius.
