# Author Correction: Diffraction-limited ultrabroadband terahertz spectroscopy

**DOI:** 10.1038/s41598-020-62995-9

**Published:** 2020-04-20

**Authors:** M. Baillergeau, K. Maussang, T. Nirrengarten, J. Palomo, L. H. Li, E. H. Linfield, A. G. Davies, S. Dhillon, J. Tignon, J. Mangeney

**Affiliations:** 1 Laboratoire Pierre Aigrain, Ecole Normale Supérieure, CNRS (UMR 8551), Université P. et M. Curie, Université D. Diderot, 75231 Paris, Cedex 05 France; 2 School of Electronic and Electrical Engineering, University of Leeds, Woodhouse Lane, Leeds, LS29JT UK

Correction to: *Scientific Reports* 10.1038/srep24811, published online 04 May 2016

The Supplementary Information file is missing from this Article and can be found below.

## Supplementary Information

Supplementary Information

