# Supplementary material for: Author Correction: Diffraction-limited ultrabroadband terahertz spectroscopy
Source: Sci Rep. 2020 Apr 20;10:6892. doi: 10.1038/s41598-020-62995-9 (PMC7170850; doi:10.1038/s41598-020-62995-9)
Supplement: Supplementary file 1 — Supplementary Information [file 41598_2020_62995_MOESM1_ESM.pdf]

SUPPLEMENTARY INFORMATION

Diffraction-limited ultrabroadband terahertz spectroscopy

M. Baillergeau[1], K. Maussang[1], T. Nirrengarten[1], J. Palomo[1], L. Li[2], E. H. Linfield[2], G. David[2], S. Dhillon[1], J. Tignon[1], J. Mangeney[1*]

[1] *Laboratoire Pierre Aigrain, Ecole Normale Supérieure, CNRS (UMR 8551), Université P. et M. Curie, Université D. Diderot, 75231 Paris Cedex 05, France*

[2]*School of Electronic and Electrical Engineering, University of Leeds, Woodhouse Lane, Leeds LS29JT, UK*

[*]juliette.mangeney@lpa.ens.fr

1. Modeling

In the following, the propagation properties of the THz radiation are modeled for a large area PA emitter, but because of the general nature of our calculations, the main conclusions drawn here are also valid for other types of large and uniform THz emitters that convert optical pulse excitations into THz electric field, such as standard electro-optic crystals. In PA, a femtosecond laser pulse generates photocarriers in its active layer, which are accelerated by the static electric field created by the electrodes. Those accelerated charges radiate a THz pulse [1] in free space. In time domain, the emission is triggered by the arrival time of the optical pulses on the PA. Radiated THz pulses can be decomposed in Fourier space as a superposition of plane waves of angular frequency ω and wave vector $\boldsymbol{k}$. For a delay in excitation τ, the corresponding spectral phase shift in frequency domain is $\phi(\omega) = -\omega\tau$. Then, one can control the spectral phase of the emitted THz electric field through the control of the corresponding delay [1]. For a large-area PA located at the position z_0 along the optical system (z-axis) and τ(x,y) the relative delay of the excitation by femtosecond optical pulses, in the direction $\boldsymbol{k}$, the angular density of radiated electrical field in the direction (θ,φ) is expressed as follow: $\frac{d^2E}{d\theta d\phi} \propto \iint A(x,y)e^{-i(\omega\tau(x,y)+kx sin\theta cos\varphi+ky sin\theta sin\varphi)}\,dxdy$ where A(x,y) is the local amplitude density of the emission of the PA for the corresponding angular frequency (proportional to the intensity of the optical pump in a first approach). This expression might be interpreted by a classical diffraction pattern of a mask with a complex transmissivity of $A(x,y)e^{-i\omega\tau(x,y)}$. Then, similarly to diffractive optics, it is possible to control the radiation pattern of the antenna by adjusting the local amplitude, which is controlled by appropriate beam shaping of the optical pulse intensity profile (i.e. A(x,y)) and a spatially dependent delay τ(x,y). The propagation of electromagnetic waves in free space is then governed by linear equations. As a consequence, the further propagation of the THz pulse is fully determined by the wavefront imposed by the optical excitation, i.e. the spatiotemporal profile of the excitation pulse.

In our configuration (see Fig. 1), the laser beam is focused before the PA (point O on the figure). If the distance z_0 between O and the PA is greater than the Rayleigh length of the laser, one can consider a conical behavior of the laser beam in the vicinity of the PA. Since the THz electric field amplitude is proportional to the intensity of the laser, its beam radius w_{THz} at 1/e on the antenna is equal to $w_{THz} = w_{opt}(z_0)/\sqrt{2}$. In order to have a physical understanding of this new excitation scheme, let us consider a point M on the antenna located at the distance w_{THz} of the center O' of the antenna. The optical pulse triggers the THz emission in M with a delay $\tau = MN/c$ after the THz emission in O'. In frequency domain, such a delay corresponds to a phase shift of the THz emission equal to $\varphi(M) = \omega_{THz}\tau = \omega_{THz}\, MN/c = k_{THz}.MN$ where k_{THz} is the THz wavevector. Furthermore, for divergence angles of the optical laser of 0.25 rad as used in our experiment: $OM = \sqrt{z_0^2 + w_{THz}^2} \approx z_0(1 + w_{THz}^2/(2z_0^2))$. We can therefore rewrite the phase shift in M : $\varphi(M) \approx k_{THz}\, w_{THz}^2/(2z_0^2)$. In our case, z_0=998µm.

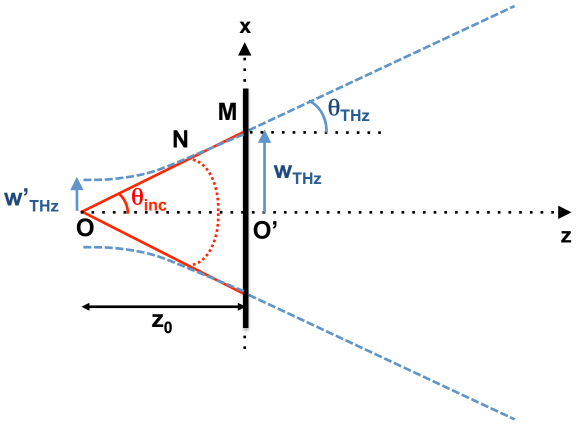

Figure 1 Schematic of the concept of spherical wavefront optical excitation of the THz emitter.

If $k_{THz}\, w_{THz}^2/(2z_0^2) << 1$, which corresponds to a THz wavelength $\lambda_{THz} \gg \lambda_c = \pi\, w_{THz}^2/z_0$, the relative phase shift between THz emission in M and O' is negligible on the surface on the PA. Thus, the excitation is the same as the one obtained under plane-wavefront optical excitation. The source of the THz Gaussian beam is located on the surface of the PA.

If $k_{THz}\, w_{THz}^2/(2z_0^2) \gg 1$, which corresponds to a THz wavelength $\lambda_{THz} \ll \lambda_c = \pi\, w_{THz}^2/z_0$, the effect of the curvature of the optical beam has to be taken into account. The THz divergence angle is the same as the divergence angle of the optical intensity. The THz radiation is a Gaussian beam with a beam waist in O, i.e. at the same position as the optical beam waist. This THz beam would be exactly the same as the one obtained from a THz Gaussian source located in O. In order to determine the virtual waist radius w'_{THz} of this THz beam, we can use the properties of the Gaussian beams: $\tan\theta = \lambda_{THz}/(\pi w'_{THz})$. Since $\tan\theta_{inc} = \lambda_{opt}/(\pi w_{opt})/\sqrt{2}$ and $\theta_{THz} = \theta_{inc}$ then $w'_{THz} = (\lambda_{THz}/\lambda_{opt})\, w_{opt}/\sqrt{2}$.

2. Experimental set-up and the photoconductive emitter.

The TDS setup uses a 15 fs, 80 MHz repetition rate pulse train centered at a wavelength of 800 nm delivered by a Ti:sapphire oscillator and a pulse compressor. The optical pulses are split into two arms for the pump and probe beams. Optical pump pulses excite the large-area PA that emits THz pulses. The THz radiation emitted by the large-area PA is backward collected (collection in a reflection geometry) to avoid absorption and dispersion of the THz pulses in the GaAs wafer. Moreover, the optical pump is incident with an angle of 45° on the PA to reduce reflection owing to lower Fresnel coefficient compared to normal incidence but also to prevent from the use of a collecting parabolic mirror with hole. The THz pulses are guided using four off-axis parabolic mirrors providing focusing of the THz radiation at the

sample position and at the detector position. The first parabolic mirror that collects THz radiation possesses a f-number of 2 and its numerical aperture is 0.25, a value equal to the numerical aperture of the parabolic mirror focusing the optical pump beam onto the antenna. The second parabolic mirror that focuses the THz beam onto the sample possesses a f-number of 2. The last parabolic mirror that focuses the THz beam onto the electro-optic crystal possesses a f-number of 1. Optical probe pulses coherently detect the focused THz radiation emitted by the antenna using conventional electro-optic detection technique [2]. We use 20-µm thick ZnTe crystal as electro-optic crystal and the probe beam is focused into the crystal with a typical spot size ranging from 85 µm to 350 µm.

The large-area PA device consists in interdigitated electrodes processed by optical lithography, which ensure strong electric field emission (owing to a large illumination area) under low electric bias (owing to small electrode spacing) [3]. The interdigitated metallic electrodes (Cr-Au) of 2 µm finger width and 2 µm spacing are deposited on 1 µm-thick LTG GaAs material grown on semi-insulating GaAs wafer. The LTG GaAs active layer associates 700 fs carrier lifetime with relatively good carrier mobility. The metal-semiconductor-metal finger structure is masked every second period by a second metallization (Cr-Au), ensuring the generation of only in-phase radiation. The second metallization is isolated from the first by a SiO_2 layer of 560 nm thickness. The antenna surface is 500×500 μm^2.

---

[1] Hu, B. B., Darrow, J. T., Zhang, X.-C., Auston, D. H. & Smith, P. R. Optically steerable photoconductive antennas. *Appl. Phys. Lett.* **56**, 888-890 (1990).

[2] Lee, Y.S. Principles of THz Science and Technology. Chap. 3, Springer, (2009).

[3] Dreyhaupt, A., Winnerl, S., Dekorsy, T., & Helm, M., High-intensity terahertz radiation from a microstructured large-area photoconductor. *Appl. Phys. Lett.* **86**, 121114-121116 (2005).
